# HS1, a Lyn Kinase Substrate, Is Abnormally Expressed in B-Chronic Lymphocytic Leukemia and Correlates with Response to Fludarabine-Based Regimen

**DOI:** 10.1371/journal.pone.0039902

**Published:** 2012-06-29

**Authors:** Federica Frezzato, Cristina Gattazzo, Veronica Martini, Valentina Trimarco, Antonella Teramo, Samuela Carraro, Anna Cabrelle, Elisa Ave, Monica Facco, Renato Zambello, Elena Tibaldi, Anna Maria Brunati, Gianpietro Semenzato, Livio Trentin

**Affiliations:** 1 Venetian Institute of Molecular Medicine (VIMM), Centro di Eccellenza per la Ricerca Biomedica, Padua, Italy; 2 Department of Medicine, Hematology and Clinical Immunology Branch, Padua University School of Medicine, Padua, Italy; 3 Department of Molecular Medicine, University of Padua, Padua, Italy; University of Barcelona, Spain

## Abstract

In B-Chronic Lymphocytic Leukemia (B-CLL) kinase Lyn is overexpressed, active, abnormally distributed, and part of a cytosolic complex involving hematopoietic lineage cell-specific protein 1 (HS1). These aberrant properties of Lyn could partially explain leukemic cells’ defective apoptosis, directly or through its substrates, for example, HS1 that has been associated to apoptosis in different cell types. To verify the hypothesis of HS1 involvement in Lyn-mediated leukemic cell survival, we investigated HS1 protein in 71 untreated B-CLL patients and 26 healthy controls. We found HS1 overexpressed in leukemic as compared to normal B lymphocytes (1.38±0.54 *vs* 0.86±0.29, p<0.01), and when HS1 levels were correlated to clinical parameters we found a higher expression of HS1 in poor-prognosis patients. Moreover, HS1 levels significantly decreased in *ex vivo* leukemic cells of patients responding to a fludarabine-containing regimen. We also observed that HS1 is partially localized in the nucleus of neoplastic B cells. All these data add new information on HS1 study, hypothesizing a pivotal role of HS1 in Lyn-mediated modulation of leukemic cells’ survival and focusing, one more time, the attention on the BCR-Lyn axis as a putative target for new therapeutic strategies in this disorder.

## Introduction

The intracellular signalling cascades involving protein tyrosine kinases of Src family (SFK) has been largely investigated in the last few years. The family consists of eight members (Lyn, Hck, Lck, Blk, Src, Fyn, Yes and Fgr) involved in signalling networks regulating metabolism, viability, proliferation, differentiation and migration of different cell types [1]–[3]. In particular, Lyn plays a key role in many signalling pathways as the most relevant SFK in B cells [4], [5]. Following antigen ligation to B-cell receptor (BCR), Lyn phosphorylates the immunoreceptor tyrosine activation motifs (ITAMs) of Igα and Igβ leading to the activation of Syk, which phosphorylates several substrates, which, in turn, activate downstream signalling molecules, including Akt, ERK, JNK, p38 MAPK, NF-AT, NF-κB [6], [7] and actin-binding proteins [8]. In human diseases, Lyn is involved in treatment resistance and progression of chronic myeloid leukemia [9], its decrease affects BCR signalling in systemic lupus erythematosus [10] and also chronic and acute leukemia subtypes showed aberrations of Lyn expression [11]. In B-cell chronic lymphocytic leukemia (CLL) Lyn kinase is overexpressed, anomalously distributed and constitutively active [12], it is a part of an aberrant cytosolic complex of 600 kDa where it is associated to one of its substrate, i.e. hematopoietic lineage cell-specific protein 1 (HS1) [13].

The 79 kDa intracellular protein HS1 [14] undergoes a process of sequential phosphorylation synergistically mediated by Syk and Lyn [15]. HS1 structure includes an Arp2/3 complex binding domain, a tandem repeats and a coil-coiled region both of which bind F-actin [16], a proline rich and a C-terminal SH3 domains [15]. HS1 also contains a region accounting for the binding with the mitochondrial protein HAX-1 [17] and a nuclear localization signal (NLS) [18]. Studies on knock-out mice highlighted HS1 as a key molecule in cell signal transduction following the BCR engagement. The lack of HS1 in B and T cells contributes to a defective proliferation and antigen receptor induced apoptosis [19]. In addition, HS1 can interact with actin and Arp2/3 complex [20], being involved in cytoskeleton modifications [16] and in the assembly of actin filaments for antigen presentation mechanism or immunological synapse formation [21]. Recently, the meaning of HS1 phosphorylation has also been studied in leukemic lymphocytes from CLL: in poor prognosis patients HS1 phosphorylation resulted to be constitutive, while in patients with good prognosis the fraction of phosphorylated protein is reduced [22]. HS1 has been reported to be involved in the apoptosis of different cell lines [20]–[24] and this capability is matter of interest since CLL has been viewed as a disease characterized by a defective apoptosis [25], [26].

The aberrant properties of Lyn contribute to the defective apoptosis of leukemic cells [12], [13] but the mechanism sustaining CLL cells survival is still unclear. In this study we focus our attention on the Lyn substrate/partner HS1 since further knowledge of this protein might be useful to understand whether Lyn alone or in association to other downstream molecules is involved in leukemic cells accumulation. To this purpose, we investigated the levels and the role of HS1 in 71 untreated CLL patients and 26 healthy controls. Our results showed that HS1 is overexpressed in leukemic cells and correlated with CLL negative prognostic factors, as well as it has an anomalous distribution in cell compartments. In addition, HS1 correlates with response to fludarabine-based therapy. All these findings could add new information on the pathogenesis of CLL and might contribute to define the BCR-Lyn-HS1 axis as a potential target for therapy in CLL.

## Results

### HS1 is Overexpressed in Leukemic Cells from Chronic Lymphocytic Leukemia Patients

**Figure 1:**
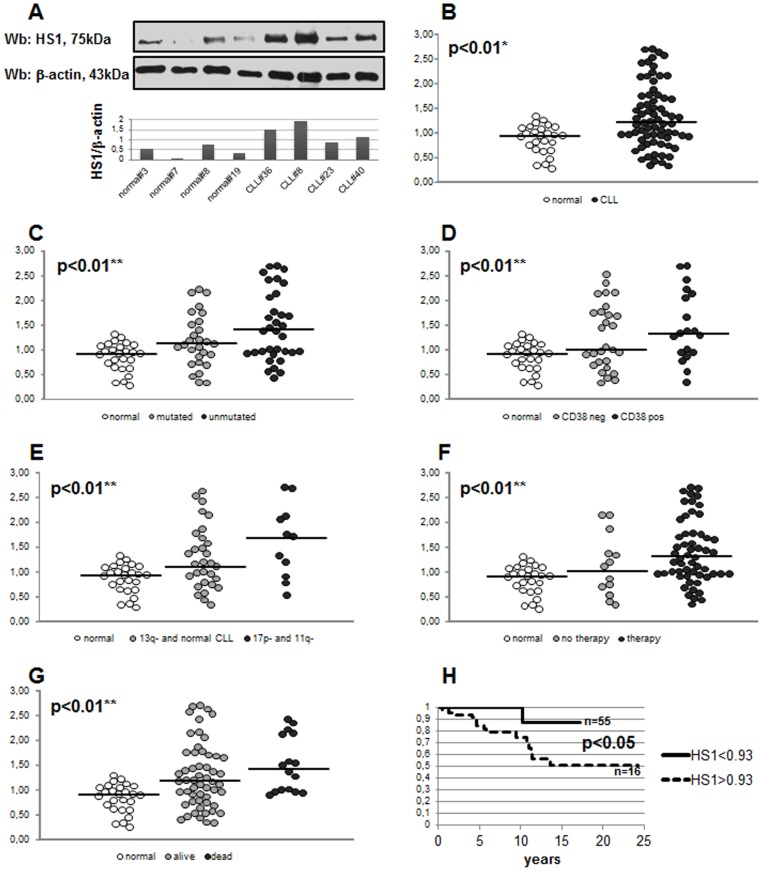
Expression of HS1 protein in CLL B lymphocytes. The lysates obtained from normal B lymphocytes and leukemic B cells from CLL patients were analyzed by immunostaining with antibody against HS1. Blots were reprobed with anti-β-actin antibody as loading control. Figure 1A is representative of four CLL and four healthy subjects with the respective densitometry of HS1/β-actin ratio. Figure 1B shows HS1/β-actin ratio of 71 CLL patients and 26 normal controls. Data has been normalized putting equal to 1 the ratio calculated in Jurkat cell line. Data obtained were evaluated for their statistical significance with the Student’s *t*-test (* p<0.01 between normal controls and CLL patients, **B**) or ANOVA (** p<0.01 between normal *vs* mutated CLL *vs* unmutated CLL, **C**; normal *vs* CD38 neg CLL *vs* CD38 pos CLL, **D**; normal *vs* 13q- and normal karyotype CLL *vs* 17p- and 11q- CLL, **E**; normal *vs* treated CLL *vs* untreated CLL, **F**; normal *vs* still alive patients *vs* dead patients, **G**). Medians are represented by solid lines. Figure 1H represents the overall survival comparison between patients (n = 16) presenting high levels of HS1 (HS1>0.93, dotted line) and patients (n = 54) presenting low levels of HS1 (HS1<0.93, solid line); the difference between curves is statistically significant (p<0.05, Kaplan Meier). 0.93 is the median of HS1 levels in normal controls.

**Figure 2:**
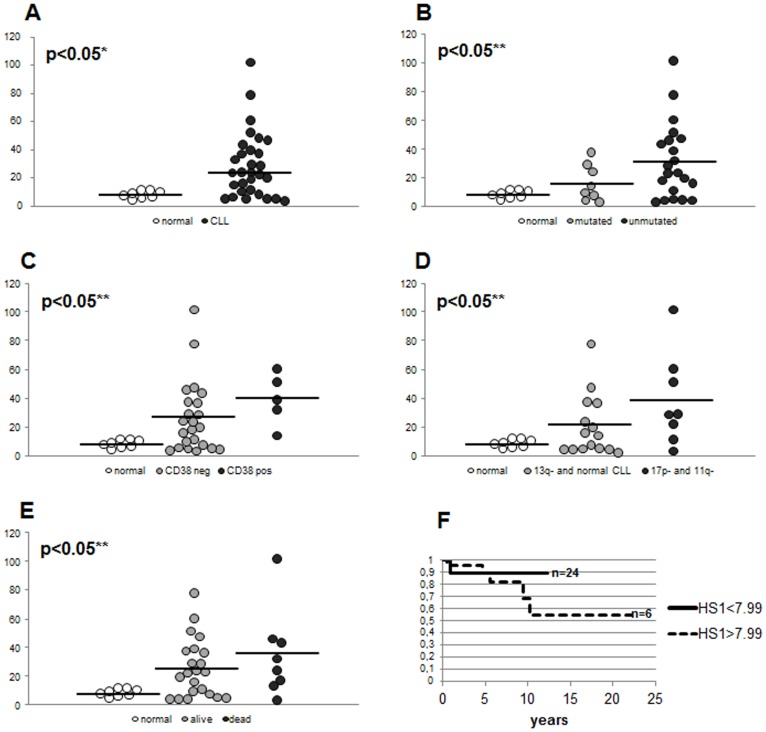
Expression of HS1 mRNA in CLL B lymphocytes. RNA extracted from normal B lymphocytes and leukemic B cells from CLL patients were analyzed for HS1 expression and normalized on GAPDH. Figure 2A shows HS1 mRNA expression of 30 CLL patients and 8 normal controls. Data obtained were evaluated for their statistical significance with the Student’s *t*-test (* p<0.05 between normal controls and CLL patients, **A**) or ANOVA (** p<0.05 between normal *vs* mutated CLL *vs* unmutated CLL, **B**; normal *vs* CD38 neg CLL *vs* CD38 pos CLL, **C**; normal *vs* 13q- and normal karyotype CLL *vs* 17p- and 11q- CLL, **D**; normal *vs* still alive patients *vs* dead patients, **E**). Medians are represented by solid lines. Figure 2F represents the overall survival comparison between patients (n = 8) presenting high levels of HS1 mRNA (HS1>7.99, dotted line) and patients (n = 26) presenting low levels of HS1 mRNA (HS1<7.99, solid line); 7.99 is the median of HS1 levels in normal controls.

HS1 protein was evaluated by western blotting analysis in B cell samples obtained from 71 untreated CLL patients and 26 healthy subjects. Figure 1A shows a representative western blotting of four CLL and four healthy subjects with the respective HS1/β-actin ratio by densitometry. Although there was considerable variation of HS1 levels in CLL cells (ranging from 0.33 to 2.70), a significant difference in HS1 expression was observed between malignant B cells from patients (1.38±0.54) and B lymphocytes from healthy subjects (0.86±0.29, p<0.01; Figure 1B). Moreover we analysed whether HS1 levels correlated with clinical parameters, in particular, with the presence or absence of somatic hypermutation (SHM), CD38 expression and cytogenetic abnormalities. We observed a high expression of HS1 in patients with negative prognostic factors (lack of SHM, CD38 positivity and presence of 17p- or 11q- aberrations). Specifically HS1 was overexpressed in unmutated (1.46±0.70) *vs* mutated patients (1.18±0.29) and healthy subjects (0.86±0.29, p<0.01; Figure 1C); in CD38 positive (1.47±0.71) *vs* CD38 negative patients (1.26±0.66) and healthy subjects (p<0.01, Figure 1D); in 17p- and 11q- (1.59±0.65) *vs* normal karyotype and 13q- patients (1.29±0.64) and healthy subjects (p<0.01, Figure 1E). In this latter analysis we did not consider 12+ aberration due to the low number of patients (n = 3, two mutated and one unmutated) showing this chromosome abnormality. We also correlated HS1 levels to the fact that patients required therapy or not and to patients survival. We observed an overexpression of HS1 levels in patients who needed therapy (1.40±0.64) with respect to patients who do not require therapy (1.09±0.62) and healthy controls (p<0.01, Figure 1F); we also observed high levels of HS1 in patients dying from CLL (1.47±0.56) *vs* patients still alive (1.28±0.66) and normal (p<0.01, Figure 1G). Overall survival estimated by Kaplan-Meier analysis showed a better survival in patients expressing low levels of HS1 with respect to patients with high levels of HS1 (p<0.05, the cut-off for HS1 high and low is 0.93 as median of HS1 levels in normal controls; Figure 1H). In approximately 40% of the patients investigated in this study we also assessed HS1 mRNA. The obtained results were compared to clinical parameters as performed for protein levels. We observed a significant difference in HS1 mRNA expression between leukemic B cells from B-CLL patients (26.93±23.43) and B lymphocytes from healthy subjects (8.00±2.80, p<0.05; Figure 2A). Moreover, HS1 mRNA was overexpressed in unmutated (31.83±26.30) *vs* mutated patients (16.37±12.72) and healthy subjects (8.00±2.80, p<0.05; Figure 2B), in CD38 positive (39.88±17.89) *vs* CD38 negative patients (26.45±25.06) and healthy subjects (p<0.05, Figure 2C), in 17p- and 11q- (38.78±31.88) *vs* normal karyotype and 13q- patients (20.64±21.39) and healthy subjects (p<0.05, Figure 2D). High levels of HS1 mRNA was also observed in patients dying from CLL (35.71±30.47) *vs* patients still alive (24.01±20.53) and normal (p<0.05, Figure 2E). Overall survival (Figure 2F) estimated by Kaplan-Meier analysis showed a better survival in patients expressing low levels of HS1 mRNA with respect to patients with high levels of HS1 mRNA; the p did not reach a statistically significant value, probably for the low number of cases; the trend of the curves is similar to what observed for protein levels (the cut-off for HS1 mRNA high and low is 7.99 as median of HS1 mRNA levels in normal controls).

### HS1 Protein Expression is Reduced after *in vivo* Treatment of Patients with Fludarabine and Cyclophosphamide

**Figure 3:**
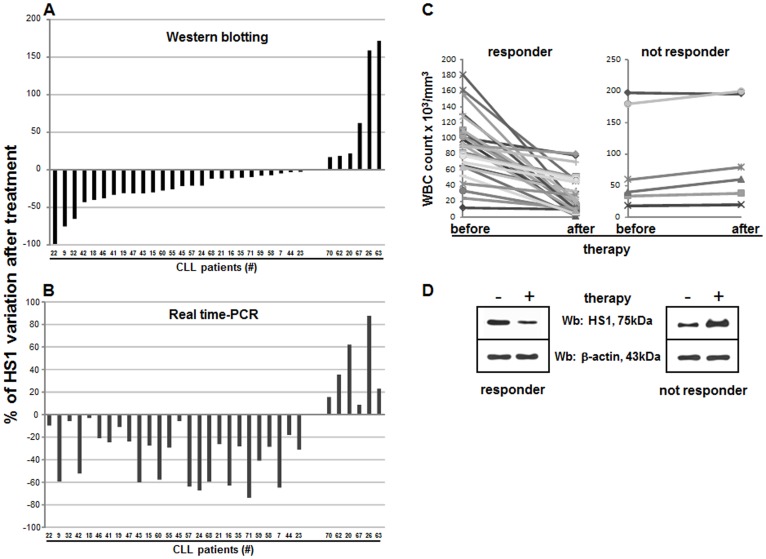
Effect of *in vivo* therapy with FLU and Cy on HS1 protein. The lysates obtained from leukemic B cells (5×10^5^ for sample) from 32 patients subject to FLU-Cy therapy were analyzed by immunostaining with antibodies against HS1 and β-actin before (-) and after (+) the administration of FLU and Cy according to FLU-Cy protocol. The same cells were processed for RNA extraction, reverse transcription in cDNA and amplification of HS1 and GAPDH by Real-Time PCR. (**A**) Histograms represent HS1 percentage of variation measured by western blotting analysis. (**B**) Histograms represent HS1 percentage of variation measured by using Real-Time PCR. (**C**) Graphics reports WBC count before and after therapy in responsive (left) and unresponsive (right) patients; the reduction of WBC count underlines the responsiveness to therapy. (**D**) The western blot in the left panel is representative of 26 patients who responded to therapy; right panel is representative of 6 patients not responding to therapy. Cy, cyclophosphamide; FLU, fludarabine.

In 32 CLL patients treated with FLU-Cy containing regimen (see Materials and methods, FLU-Cy protocol), the levels of HS1 protein was determined in freshly isolated and purified leukemic B cells before and after three days of therapy, period corresponding to the first cycle of therapy. Figure 3A shows the percentage of HS1 protein variation by western blotting after *in vivo* treatment: some patients show a reduction of HS1 after therapy (negative values) while others displayed increase of the protein (positive values). When the expression of HS1 was assessed by Real-Time PCR (Figure 3B), the amount of variations (positive or negative) correlated with those obtained by western blotting. Interestingly, all the patients (26/32) showing a reduction of HS1 after treatment responded to therapy (marked reduction of CD19+/CD5+ clonal cells and of the number of white blood cells; Figure 3C, left panel). On the contrary, in non responding patients (6/32) who did decrease in lymphocytes number after therapy (Figure 3C, right panel) not only we did not observe any reduction (of HS1) but found an increase of HS1. Figure 3D shows western blotting analysis of a representative responding (CLL#47 of panel A) and non responding (CLL#67 of panel A) patient. In responding patients HS1 variations before and after therapy, measured with a Paired Student’s *t*-test, was highly significant (western blotting p = 0.002 and Real-Time PCR p = 0.001) while in non responding patients the variation before and after therapy was significant only in Real-Time PCR (p = 0.024, western blotting p = 0.067) probably because of the little number of non responding patients.

### HS1 Protein Decreased and Underwent Cleavage Following *in vitro* Treatment of Leukemic Cells with Fludarabine and Cyclophosphamide

**Figure 4:**
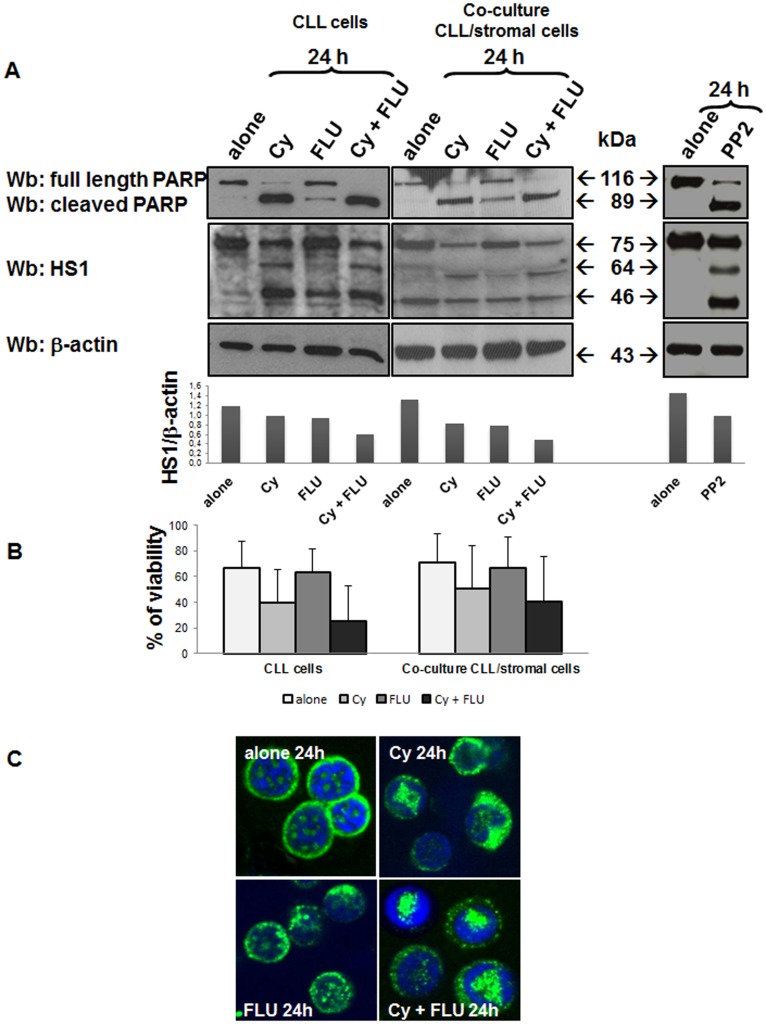
Effect of *in vitro* treatment with FLU and Cy on HS1 protein. (**A**) CLL cells were cultured for 24 hours alone or in presence of Cy (5 mM), FLU (20 µM) and Cy and FLU together (left panel), with the same drugs but in co-culture with the HS-5 stromal cell line (panel in the middle) or with PP2 (20 µM) (right panel). Subsequently, cells were processed for SDS/PAGE, transferred on nitrocellulose membrane and treated with polyclonal anti-PARP antibody, to put in evidence cell apoptosis, anti-HS1 polyclonal and anti-β-actin. Histograms represents full length HS1/β-actin ratio densitometric analysis. (**B**) Cell viability under the same conditions of point A was assessed by Annexin/PI test; histograms represent the mean±SD of percentage of cell viability of 8 CLL patients samples cultured alone (histograms on the left) or in co-culture with stromal cells (histograms on the right). (**C**) Confocal microscopy analysis of HS1 (FITC, green) in leukemic B lymphocytes cultured 24 hours alone or in presence of Cy, FLU and Cy and FLU together; nuclei were stained with DAPI (blue). UltraView LCI confocal system, UltraView LCI 5.0 acquisition software; original magnification, ×60. Cy, cyclophosphamide; FLU, fludarabine.

Several studies have demonstrated the involvement of HS1 in the apoptotic process [19], [27]. The protein is also a substrate of caspase-3, being cleaved into two fragments (64 and 46 kDa) in Jurkat cell line during Fas-induced apoptosis [20]. We have therefore studied HS1 pattern in leukemic B cells obtained from 8 CLL patients cultured in medium alone and in the presence of pro-apoptotic substances. In particular, we performed experiments with the same compounds (FLU and Cy) utilized for therapy. After 24 h incubation, we showed that apoptosis induction was associated to a reduction of HS1 levels with the appearance of two new fragments of 64 and 46 kDa, produced by HS1 cleavage (Figure 4A, blots on the left with the respective densitometry). Apoptosis induction is demonstrated by the presence of the cleaved form of PARP and by the pyknotic nuclei in Figure 4C. FLU and Cy together had a stronger effect on cells apoptosis with respect to single agents: cells cultured in the presence of the two compounds showed higher levels of cleaved PARP. This fact was also confirmed by the percentage of viable cells assessed, under different conditions, by Annexin V/PI test (Figure 4B, histograms on the left). Moreover, the presence of the two new cutting fragments could designate HS1 as a new apoptosis marker. In particular, the 46 kDa fragment, which appears even with a lite amount of cleaved PARP (cells incubated with FLU alone), could be indicative of an early apoptosis, while the fragment of 64 kDa, that we observed when the majority of PARP was cut (cells incubated with Cy alone or Cy in association with FLU), could be an indicator of late apoptosis. In the right panel of Figure 4A we showed the same experiment performed with PP2; the most effective and selective inhibitor for tyrosine kinases of the Src family and, in particular, of Lyn. In CLL B cells, apoptosis induced by Lyn inhibition had on HS1 the same effect of drug-induced apoptosis revealing a link between Lyn, HS1 and programmed cell death. Among patients evaluated *in vitro*, four of them (CLL#9, #24, #46, #60) were assessed before and after *in vivo* treatment (see previous point). Considering that HS1 has previously been associated with trafficking and homing of CLL cells and *in vitro* studies including stromal cells have depicted the relevance of the microenvironment protecting CLL cells from apoptosis [28], the same experiments were performed after culturing CLL B cells on a layer of the human bone marrow stromal cell line HS-5. Also in this case AnnexinV/PI test was performed to test cell viability. Data obtained demonstrated that HS-5 didn’t protect HS1 from the degradation and the cleavage induced by drugs. Cell viability (Figure 4B, histograms on the right), but also PARP and HS1 patterns (Figure 4A, blots in the middle) are the same as those observed for the cells not co-cultured.

### HS1 has a Nuclear Distribution in Leukemic B Cells

**Figure 5:**
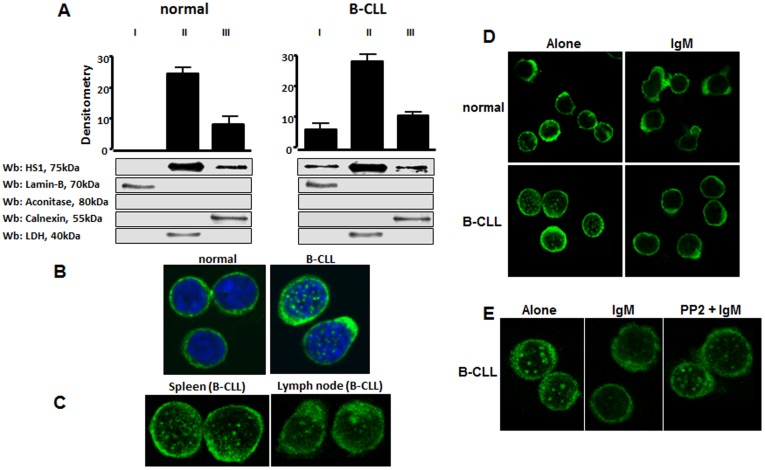
Subcellular localization of HS1 in normal and CLL B cells. (**A**) Analysis by differential ultracentrifugation. B cells were sonicated in isotonic buffer and nuclei (I), cytosol (II) and microsomes (III) were separated by ultracentrifugation. Comparable aliquots of the different fractions were loaded on SDS/PAGE and the separated proteins were immunostained with anti-HS1 antibody, anti-LDH (cytosolic marker), anti-calnexin (endoplasmic reticulum marker), anti-PMCA (plasma membrane marker), anti-lamin-B (nuclear marker) and anti-aconitase (mitochondrial marker) antibodies. Figure is representative of different experiments performed on 10 CLL patients and 5 normal controls. The diagram shows the mean ± SD of HS1 of all the samples examined. (**B**) Confocal microscopy analysis of HS1 (FITC, green) in normal and leukemic B lymphocytes obtained from peripheral blood; nuclei were stained with DAPI (blue). The analysis of HS1 immunolocalization was performed in 6 different normal and 15 CLL samples. (**C**) Confocal microscopy analysis of HS1 (FITC, green) in leukemic cells from spleen and lymph node of a CLL patient. (**D**) Confocal microscopy analysis of normal and leukemic B lymphocytes after incubation alone or with anti-human IgM (10 µg/ml) for 10 minutes at 37°C for BCR activation. Cells were immunostained with anti-HS1 antibody (FITC, green). (**E**) The same experiment of point D was performed in CLL lymphocytes pre-incubating cells with the Lyn kinase inhibitor PP2 (10 µM). The figure is representative of the experiments performed in 4 CLL patients. UltraView LCI confocal system, UltraView LCI 5.0 acquisition software; original magnification, ×60.

Using differential ultracentrifugation, subcellular localization of HS1 protein was analyzed in normal and leukemic B lymphocytes. The experiments were performed in 10 CLL patients and 5 healthy subjects. To this purpose, cells were sonicated in isotonic buffer and cytosol, microsomes and nuclei were separated from each other. HS1, which is a cytosolic protein, was only particulated and exclusively detected in the cytosolic fraction of normal B cells, while an aliquot of it (approximately 4–7% of the total protein) was also found in the nucleus of all leukemic samples examined (Figure 5A). To confirm this result, we also investigated the microscopic distribution of HS1 in malignant cells. Figure 5B shows a representative pattern of HS1 evaluated in 14 patients and 6 healthy subjects. HS1 in normal B cells is uniformly distributed in the cytosol while in CLL cells HS1 also appears with a nuclear spotting distribution. The experiment was also performed using B lymphocytes extracted from spleen and lymph node of a CLL patients and also in this case HS1 presents a nuclear distribution (Figure 5C).

### HS1 Nuclear Escape after B Cell Receptor Engagement is Mediated by Lyn Kinase

Since HS1 is commonly phosphorylated after BCR engagement [15], [19], [27], we investigated whether HS1 undergoes some changes in cell distribution after BCR triggering. To this purpose BCR was triggered with anti-IgM antibody and cells were collected, washed and mounted on poly-L-lysine-coated slides and processed for confocal microscopy. We observed that HS1 disappeared from the nucleus in leukemic cells following culture in the presence of anti-IgM (Figure 4D). The pre-incubation of leukemic cells with PP2, a Src kinase inhibitor, abolished the IgM-mediated HS1 redistribution (Figure 5E). The disappearance of nuclear HS1 can be ascribed to Src kinases which can act at an upstream level of the signal cascade. Considering that Lyn is key Src kinase in CLL [12], it can be assumed that Lyn itself mediates HS1 redistribution.

## Discussion

In this study we demonstrated that HS1, a Lyn substrate, is overexpressed in leukemic cells from CLL patients as compared to normal B lymphocytes and, in particular, HS1 levels correlate with poor prognosis. Furthermore, HS1 is *in vivo* down-regulated by FLU-Cy therapy and is abnormally expressed in leukemic cells, being observed in the nucleus.

We previously found that Lyn kinase in CLL sustains the survival of leukemic cells, suggesting a role for this kinase in CLL pathogenesis and identifying this tyrosine kinase as a potential therapeutic target [12], [13]. Since the mechanism by which Lyn acts on the survival of the malignant clone is still unclear, we investigated the Lyn partner/substrate HS1. HS1 is phosphorylated by Lyn [15] and is a partner of Lyn in the aberrant complex [13].

First of all we demonstrated that HS1 is overexpressed in leukemic with respect to normal B cells (p<0.01). Considering that CLL patients can be grouped into different subsets on the basis of biological features that have been shown to predict the clinical behaviour of their disease we then correlated the levels of HS1 to the presence or absence of adverse prognostic factors observing a pattern of HS1 that increased with the severity of the disease. Despite the broad heterogeneity among CLL samples, our data demonstrated a relationship between HS1 protein expression and prognostic factors: high HS1 expression was associated with lack of IgVH mutations [29], positivity to CD38 [30] and presence of genetic abnormalities such as 17p- and 11q- [31]. Patients presenting IgVH mutations, negativity to CD38 and a normal or 13q- kariotype had levels of HS1 which were close to normal subjects levels. In our analysis HS1 didn’t correlate to ZAP70 expression [32].

Although it is pretentious to consider HS1 levels as a predictor of overall survival, its expression correlated with patients’ survival as demonstrated by Kaplan-Meier plot (p<0.05, Figure 1H). Moreover, although this type of analysis didn’t reach full statistical significance (p = 0.052, Kaplan-Meier plot), the trend in the levels of HS1 also suggests its correlation with time to first treatment: patients with high levels of HS1 require treatment before with respect to patients with low levels of this proteins (data not shown). HS1 mRNA analysis performed in 30/71 patients of this study correlated to data obtained by protein analysis.

Taken together our data converge to assign to HS1 a prognostic relevance in CLL as, indeed, had already been anticipated by Scielzo C. et al. concerning HS1 phosphorylation (patients with poor prognosis are characterized by an important expression of the phosphorylated form of HS1) [22].

When we analyzed HS1 in patients following FLU-Cy therapy, we found that both HS1 mRNA and protein were tightly regulated over time by therapy. HS1, infact, decreased after treatment only in patients responsive to therapy.

Overexpression of HS1 in leukemic B cells and its down-regulation by an effective treatment allowed us to hypothesize that HS1 might be involved in the maintenance of neoplastic B cell survival. In unresponsive patients, HS1 is not down-modulated by therapy *in vivo*, suggesting its putative involvement in the chemoresistance of these patients. It might be hypothesized that these results may indicate that an up-regulation of HS1 could counteract the cytotoxic effects of drugs in non responding patients, similarly to what was observed in patients with p53 mutations [33] and/or Mcl-1 overexpression [34].

The fact that HS1 is directly involved in cell death mediated by drugs is consistent with several studies providing evidence of HS1 involvement in programmed cell death [19], [20], [27]. Our results obtained by incubating leukemic cells with pro-apoptotic drugs, support the observation that HS1 is involved in leukemic cell survival. In particular, the *in vitro* incubation of leukemic cells with FLU and Cy induced a reduction of HS1 level and the appearance of two new cleavage fragments, which are commonly produced following apoptotic process in Jurkat cell line [20]. Whether HS1 protein has a specific function or one of its fragments have different physiological roles or simply represent a byproduct of protein degradation is still undefined. What we know is that *in vitro* reduction and cleavage of HS1 is mediated by caspases activation since this action is prevented by the caspases inhibitor Z-VAD fmk (data not shown). The experiment performed using the Lyn inhibitor PP2 led to the same result. It is demonstrated that the inhibition of Lyn activity obtained by treating the leukemic cells with specific inhibitors (PP2 in this case) is sufficient to restore cell apoptosis, providing a correlation between high basal Lyn activity and defects in the induction of the programmed cell death in CLL B cells [12]. The effect of Lyn inhibition on its substrate HS1 is the same of Flu and Cy treatment demonstrating that Lyn inhibition or, better, apoptosis induced by Lyn inhibition is involved in the cleavage of HS1. This established a link between HS1, Lyn and programmed cell death.

Another issue of this study was to characterize HS1 distribution inside the cell in terms of subcellular localization.

The subcellular analysis of HS1 showed that this molecule is abnormally distributed in malignant cells as compared to normal B cells; while in normal B lymphocytes HS1 is localized only in the cytosol, in neoplastic B cells it is detected both in the cytosol and in the nucleus where it has a spotting distribution. Interestingly, the observation that HS1 structure presents a NLS motif [27] and shares a high amino-acid sequence homology to some transcriptional factors, might suggest that the protein may be involved in the regulation of gene expression, that could explain its presence in the nucleus [14]. The fact that HS1 is found also in the nucleus of leukemic and not of normal B cells, could suggest a putative role for HS1 itself in the regulation of genes responsible of leukemic cell survival. In addition, HS1 disappeared from the nucleus when cells triggered by anti-human IgM [19]. Our data suggest that, in leukemic B cells, HS1 moves from the nucleus to the cytoplasm after BCR engagement and this escape is prevent by the Lyn inhibitor PP2 action. This observation further points out the link between Lyn and its substrate HS1 highlighting how the redistribution of this protein within the cell could be mediated upstream by Lyn. Data from the literature on WEHI-231 cells reported that, after BCR triggering, Lyn and Syk kinases phosphorylate HS1 that migrates to the nucleus and mediates apoptosis [19], [27]. In our study, following BCR stimulation, HS1 leaves the nucleus where it is abnormally present. These different results might be further investigated considering that in the literature HS1 behavior is described to be exactly the opposite [19], [27], related to the different cell types used.

In conclusion, HS1 is involved in the survival of the neoplastic clone; it remains still unclear whether HS1 only provides an inhibition of apoptosis or otherwise increases cell proliferation, or is equipped with both activities. Our data endorsed an important role for HS1 in survival maintenance of malignant B cells of CLL. For this reason, HS1 could represent a new target for a targeted therapy in order to block the proliferation of leukemic cells and make them responsive to drug-induced apoptotic stimuli. The suggestion of a putative therapeutic use of HS1 once again focuses the attention on BCR-Lyn-HS1-cellular activation and apoptosis resistance pathways. Both Lyn and HS1 could represent therapeutic targets to make leukemic cells more responsive to drugs-induced pro-apoptotic stimuli during the treatment of CLL patients.

## Materials and Methods

### Ethics Statement

Written informed consents were obtained from all patients, prior to sample collection, according to the Declaration of Helsinki. The ethic approval for our study was obtained from the local ethic committee of “Regione Veneto on chronic lymphocytic leukemia”.

### Patients, Cell Separation and Culture Conditions

Blood samples were collected from 71 patients that satisfied standard morphologic and immunophenotypic criteria for CLL B cells. Patient characteristics are reported in Table 1. All specimens were collected from patients attending the Hematology and Clinical Immunology Branch, Padova University School of Medicine from 2003 to 2008. At the time of the collection, patients had never received treatment; the interval between collection and treatment ranged from 3 to 12 months. Peripheral blood mononuclear cells (PBMCs) were separated by Ficoll gradient centrifugation (Amersham Biosciences; Buckinghamshire, UK). When necessary, B cells were purified from PBMCs by removing T cells by rosetting method with sheep erythrocytes. The samples that were used had at least 95% CD5+/CD19+ cells, as assessed by flow-cytometry. Normal B cells from 26 Buffy Coat, representative of the adult healthy population, were isolated by negative selection using the RosetteSep for B cells isolation kit (Stemcell Technologies; Vancouver, Canada). The purity of the obtained B cells was at least 95% (CD19+), as assessed by flow cytometry. In some experiments purified B cells (2×10^6^ cells/ml) were cultured in RPMI-1640 medium supplemented with 10% heated inactivated fetal calf serum, 2 mM L-glutamine, 100 U/ml penicillin, and 100 µg/ml streptomycin (Invitrogen; Paisley, UK), at 37°C in a humidified atmosphere containing 5% CO_2_, with fludarabine (FLU, Fludara, Schering; Berlin, Germany) and cyclophosphamide (Cy, Sigma-Aldrich; Milano, Italy) or with goat F(ab')2 anti-human IgM (10 µg/ml) (Invitrogen; Paisley, UK) and PP2 (Calbiochem; Gibbstown, NJ). In preliminary experiments, the dose-response of the CLL cell cultures to the different compounds was analyzed and the concentration required to induce death in 50% of leukemic cells (IC50) was determined. The IC50 values for PP2 and FLU were 10 µM and 20 µM respectively. For cyclophosphamide a dose-response curve was performed and the dose of 5 mM has been chosen to achieve the maximal apoptotic effect. In other experiments we have used B cells obtained from 32 CLL patients before and after therapy with FLU and Cy according to the protocol FLU-Cy: FLU 25 mg/m^2^ for 3 days and Cy 350 mg/m^2^ for 3 days, every 28 days; this therapy is the standard of care for patients needing a therapy. For co-culture experiments, 1×10^5^/well irradiated HS-5 (a human bone marrow stromal cell line) cells were seeded into 12 well plates and CLL B cells were added at a ratio of 4∶1.

**Table 1:** Biological and clinical characteristic of patients.

Patients	71
Median age, years (range)	64 (40–95)
Male/Female	41/30
Wbc count,/mm^3^ (range)	61209 (8300–198200)
Lymphocytes, % (range)	80 (41–97)
Mutated^1^	28
ZAP70 positive^2^	30
CD38 positive^3^	20
Karyotype (N^4^/13q−/12+/11q−/17p-)	12/18/3/7/4

1 “Mutated” was defined as having a frequency of mutations greater than 2% from germline V_H_ sequence.

2 As determined by cytofluorymetric analysis (cut-off: 20%).

3 As determined by cytofluorymetric analysis (cut-off: 30%).

4 N =  normal karyotype.

### HS1 Protein Expression

Cells (5×10^5^ for each assay) were prepared by cell lyses with Tris 20 mM, NaCl 150 mM, EDTA 2 mM, EGTA 2 mM, Triton X-100 0.5% supplemented with complete protease inhibitor cocktail (Roche; Mannheim, Germany) and sodium orthovanadate 1 mM (Calbiochem; Gibbstown, NJ). Samples were then subjected to SDS/PAGE (10% gels), transferred to nitrocellulose membranes, and immunostained with anti-HS1 monoclonal antibody (Becton Dickinson; Franklin Lakes, NJ) and anti-β-actin monoclonal antibody (Sigma-Aldrich; Milano, Italy) using an enhanced chemiluminescent detection system (Pierce; Rockford, IL). For the *in vitro* apoptosis study membranes were stained in succession with anti-PARP polyclonal antibody (Cell Signaling Technology, Inc.; Danvers, MA), anti-HS1 polyclonal antibody (produced in A.M. Brunati laboratory) and anti-β-actin monoclonal antibody.

### HS1 mRNA Expression by Real-Time PCR Analysis

Total cellular RNA from patient samples were extracted from 5–10×10^6^ leukemic cells freshly isolated or before and after therapy using RNeasy Mini Kit (Qiagen; Hilden, Germany), according to the manufacturer's protocol and treated with DNase (Qiagen). First strand complementary DNA (cDNA) was generated from 1 µg total RNA using oligo-dT primer and the AMV reverse transcriptase (Reverse Transcription System, Promega Corporation; Madison, WI). Real-Time quantitative PCR amplifications reaction were carried out in an ABI Prism 7000 sequence detection system (Applied Biosystems; Foster City, CA) in a 15 µl volume. SYBR Green PCR Master Mix was purchased from Applied Biosystems (P/N 4309155), containing AmpliTaq Gold DNA Polymerase and optimized buffer components. A fraction of 5 µM primers and 1,5 µl of cDNA were added to SYBR Green master mix to make a final 15 µl reaction volume. The primers used for HS1 and GAPDH amplifications are: HS1: Forward 5′- GTG AGA ACC AGC AGG GAA CAC -3′ Reverse 5′- CAT TGT CCT CCG GGA GAG TCT -3′; GAPDH: Forward 5′- AAT GGA AAT CCC ATC ACC ATC T -3′ Reverse 5′- CGC CCC ACT TGA TTT TGG -3′. These primers were obtained using the Primer Express computer software (Applied Biosystems). PCR reactions were performed under the following conditions: initial denaturation at 95°C for 10 min followed by 95°C for 15 s and 60°C for 1 s cycled 50 times. Each quantitation target was amplified in duplicate samples. A no template control for each master mix and two standard curves were generated for GAPDH and HS1 using Jurkat cDNA in a serial dilution 1, 1∶5, 1∶25 and 1∶125. The relative amounts of mRNA was determined by comparison with standard curves. For each sample, results were normalized for GAPDH expression. To distinguish specific amplicons from non-specific amplifications, a dissociation curve was generated.

### Cell Viability Testing

Apoptosis was assessed using the Annexin V Apoptosis Detection Kit (Becton Dickinson). After 24 hours of incubation with FLU and Cy aliquots of 5×10^5^ cells were harvested, washed, and incubated for 10 min in the dark and at RT with 100 µl of binding buffer, 5 µl of Annexin V-FITC, and 10 µl of Propidium iodide (PI), provided by the kit (1 µl/ml final concentration). After the incubation, 100 µl of binding buffer were added and cells were analyzed by flow cytometer FACScan. For each sample 20.000 events were acquired and the number of viable cells was expressed as percentage of Annexin V/PI negative cells in the total cells analyzed.

### Confocal Microscopy Analysis

Cells were plated in polylisine coated glass for 15 min at room temperature and fixed in 4% paraformaldehyde for 10 min. The fixed cells were then washed twice with PBS 1X and permeabilized with 0.1% Triton X-100 (Sigma-Aldrich) for 4 min. Before staining, non-specific protein binding was blocked by incubating slides for at least 30 min in 2% BSA. Cells were then stained with diluted mouse monoclonal IgG1 anti-HS1 (Becton Dickinson) at least 1 h at room temperature or overnight at 4°C followed by the appropriate secondary antibody FITC conjugated. Nuclei were stained with DAPI (Invitrogen). Background staining with control antibodies was routinely compared with positively stained cells and was not visible using identical acquisition settings. Slides were mounted with cover slips and fluorescence was detected using the UltraView LCI confocal system equipped with a fluorescence filter set for excitation at 488 nm.

### HS1 Subcellular Localization

Approximately 4×10^6^ cells were sonicated in 400 µl of isotonic buffer containing 50 mM Tris/HCl (pH 7.5), 0.25 M saccharose, 1 mM orthovanadate and protease inhibitor cocktail (Boehringer; Mannheim, Germany) and centrifuged at 10 000 g for 10 min in order to separate the particulate fraction containing cell debris, nuclei and other cellular particles (pellet I). The supernatant was further centrifuged at 100 000 g for 30 min to separate the cytosol from the microsomes (pellet II). The pellets were resuspended in 400 µl of lysis buffer and 20 µl of the different fractions were loaded on SDS/PAGE, blotted and immunostained with anti-HS1, anti-LDH, anti-calnexin, anti-PMCA, anti-lamin and anti-aconitase (Santa Cruz Biotechnology; Santa Cruz, CA).

### Statistical Analysis

Statistical analysis was performed using Student’s *t* test, Paired Student’s *t* test and ANOVA. Data are reported as median ± standard deviation (SD). P value <0.05 was considered significant. Overall survival was calculated from date of diagnosis, and curves were constructed using the method of Kaplan and Meier.
